# Isolation and Characterization of a Novel Marine Peptide, WPN-15, from Walleye Pollock (*Gadus chalcogrammus*) Tail By-Products and Its Therapeutic Effects Against Atopic Dermatitis

**DOI:** 10.3390/pharmaceutics18070895

**Published:** 2026-07-21

**Authors:** Sung-Gyu Lee, Jin-Woo Hwang, Hyun Kang

**Affiliations:** 1 Department of Medical Laboratory Science, College of Health Science, Dankook University, Cheonan-si 31116, Chungcheongnam-do, Republic of Korea; sung-gyu@dankook.ac.kr (S.-G.L.); croucard@naver.com (J.-W.H.); 2 Marine Bio-Food and Drug Convergence Technology Center, Dankook University, Cheonan-si 31116, Chungcheongnam-do, Republic of Korea

**Keywords:** marine-derived peptide, *Gadus chalcogrammus*, fish by-products, STAT3 signaling, atopic dermatitis

## Abstract

**Background/Objectives**: Atopic dermatitis (AD) is a multifactorial inflammatory skin disorder in which epidermal barrier disruption and dysregulated immune responses drive persistent cutaneous inflammation. Owing to their broad spectrum of biological activities, marine-derived peptides have attracted increasing attention as potential therapeutic agents capable of modulating inflammatory and immune pathways. **Methods**: In this study, a novel peptide, WPN-15 (NGAIADQQPQRPNIV), was isolated from enzymatic hydrolysates of walleye pollock (*Gadus chalcogrammus*) tail by-products using an activity-guided purification process consisting of dialysis, fast protein liquid chromatography-gel permeation chromatography (FPLC-GPC), reverse-phase high-performance liquid chromatography (RP-HPLC), and electrospray ionization mass spectrometry (ESI-MS). The anti-inflammatory activity of WPN-15 was first examined in lipopolysaccharide (LPS)-stimulated RAW 264.7 macrophages, and subsequently validated in a 2,4-dinitrochlorobenzene (DNCB)-induced atopic dermatitis model using six-week-old male BALB/c mice. **Results**: WPN-15 significantly inhibited nitric oxide production in LPS-stimulated macrophages without causing cytotoxic effects. Topical administration of WPN-15 markedly alleviated DNCB-induced AD-like kin lesions, significantly reduced dermatitis severity scores, and decreased serum interleukin (IL)-6 levels. Histological evaluation further demonstrated that WPN-15 attenuated epidermal hyperplasia, dermal thickening, and mast cell infiltration. Furthermore, WPN-15 significantly downregulated the mRNA expression of IL-1β and IL-6 and inhibited signal transducer and activator of transcription 3 (STAT3) phosphorylation in skin tissues, indicating that its protective effects are mediated, at least in part, through the suppression of the IL-6/STAT3 signaling pathway. **Conclusions**: WPN-15 effectively attenuated inflammatory responses and pathological features associated with experimental AD. These findings demonstrate that walleye pollock tail by-products represent a valuable and sustainable source of bioactive peptides and support the potential application of WPN-15 as a marine-derived therapeutic candidate for the management of AD.

## 1. Introduction

Atopic dermatitis (AD) is a chronic immune-mediated inflammatory skin disorder characterized by recurrent episodes of pruritic eczema, epidermal barrier dysfunction, and abnormal immune activation. The disease affects individuals of all ages, with an estimated prevalence of approximately 15–20% of children and 5–10% of adults worldwide, and its incidence continues to increase in both developed and developing countries [1,2]. Beyond its cutaneous manifestations, AD is associated with substantial socioeconomic and healthcare burdens because recurrent exacerbations frequently impair patients’ quality of life and require long-term therapeutic management [3].

The pathogenesis of AD is driven by complex interactions between genetic predisposition, epidermal barrier dysfunction, environmental triggers, microbial imbalance, and immune dysfunction [4]. During disease progression, activated keratinocytes together with infiltrating immune cells release numerous pro-inflammatory mediators, including interleukin (IL)-1β, IL-4, IL-6, IL-13, thymic stromal lymphopoietin (TSLP), and tumor necrosis factor-α (TNF-α), thereby perpetuating cutaneous inflammation and contributing to pathological tissue remodeling [5,6]. Among these inflammatory mediators, IL-6 is recognized as a key regulator of both acute and chronic inflammatory processes, and elevated IL-6 levels have been closely associated with disease severity in patients with AD [7].

Accumulating evidence indicates that the Janus kinase (JAK)/signal transducer and activator of transcription (STAT) pathway plays a central role in regulating inflammatory processes associated with AD pathogenesis. Among the STAT family members, STAT3 is particularly important because its activation promotes the production of pro-inflammatory cytokines, recruitment of immune cells, epidermal hyperplasia, and impairment of skin barrier homeostasis [8,9]. Moreover, persistent activation of STAT3 has been widely observed in chronic inflammatory skin diseases, supporting the IL-6/JAK/STAT3 signaling axis as a promising therapeutic target for AD and other immune-mediated dermatological disorders [10].

Although current therapeutic strategies, including topical corticosteroids, calcineurin inhibitors, antihistamines, and biologics such as dupilumab, have markedly improved the clinical management of AD, their prolonged use is often constrained by adverse reactions, high treatment costs, and heterogeneous therapeutic responses among patients [11,12,13]. Consequently, increasing attention has been directed toward the discovery of naturally derived bioactive compounds that offer improved safety and effective therapeutic benefits.

Marine environments represent a rich reservoir of structurally diverse natural compounds with broad pharmaceutical and nutraceutical potential. Among these, marine-derived peptides have attracted considerable scientific interest owing to their diverse biological functions, including antioxidant, anti-inflammatory, antimicrobial, immunomodulatory, and wound-healing effects [14,15]. Compared with synthetic therapeutic agents, marine peptides are generally characterized by excellent biocompatibility, relatively low toxicity, and high biological selectivity, making them attractive candidates for applications in functional foods, cosmeceuticals, and pharmaceutical products [16].

Seafood processing generates substantial quantities of protein-rich by-products, such as fish heads, skins, bones, viscera, and tails, many of which remain insufficiently utilized despite their considerable nutritional and functional value [17]. The development of enzymatic hydrolysis technologies has facilitated the conversion of these underutilized materials into peptide-rich products with a variety of biological activities [18]. Previous studies have demonstrated that peptides derived from marine fish possess antioxidant, antihypertensive, anti-obesity, and anti-inflammatory properties. However, the isolation and characterization of peptides specifically targeting atopic dermatitis from fish-processing by-products have received relatively little attention [19,20].

Walleye pollock (*Gadus chalcogrammus*) is one of the world’s major commercial fish species and is widely processed for surimi and other seafood products, generating substantial quantities of tail by-products. Although these by-products represent an abundant protein resource, their application as a source of functional peptides remains largely unexplored [21,22]. Utilizing these discarded materials for bioactive peptide production not only enhances resource sustainability but also creates opportunities for developing high-value marine functional ingredients [23,24]. Whereas our previous investigation employed antioxidant activity-guided purification to isolate a bioactive peptide [25], the present study implements an anti-inflammatory activity-guided screening strategy to discover a novel peptide with therapeutic potential against AD.

Based on this rationale, the present study aims to identify anti-inflammatory peptides from enzymatically hydrolyzed walleye pollock tail by-products using an activity-guided purification approach. The isolated peptide was structurally characterized through chromatographic purification and mass spectrometric analyses, after which its anti-inflammatory efficacy was assessed in lipopolysaccharide (LPS)-stimulated RAW 264.7 macrophages and subsequently validated in a 2,4-dinitrochlorobenzene (DNCB)-induced mouse model of atopic dermatitis. Furthermore, the underlying molecular mechanism was explored by examining inflammatory cytokine expression and the activation of the STAT3 signaling pathway.

## 2. Materials and Methods

### 2.1. Experimental Materials

Fresh walleye pollock tail by-products were purchased from a local fish market in Cheonan, Republic of Korea, freeze-stored at −20 °C, and used throughout the study. Commercial food-grade proteases, including Alcalase, Flavourzyme, Neutrase, and Protamex, were supplied by Novozyme Co. (Bagsvaerd, Denmark). Unless otherwise stated, all analytical-grade reagents, including LPS (from *Escherichia coli* O111:B4), dimethyl sulfoxide (DMSO), 3-(4,5-Dimethylthiazol-2-yl)-2,5-diphenyltetrazolium bromide (MTT) reagent, Griess reagent, DNCB, and terfenadine, were obtained from Sigma-Aldrich (St. Louis, MO, USA). RAW 264.7 murine macrophages (KCLB No. 40071; Cellosaurus accession: CVCL_0493) were purchased from the Korean Cell Line Bank (KCLB, Seoul, Republic of Korea). Cell culture reagents, including Dulbecco’s Modified Eagle Medium (DMEM), fetal bovine serum (FBS), and penicillin-streptomycin solution, were purchased from Gibco (Grand Island, NY, USA). Six-week-old male BALB/c mice were supplied by DBL Co., Ltd. (Eumseong, Republic of Korea). Mouse IL-6 concentrations were determined using a commercial ELISA kit purchased from R&D Systems -(Minneapolis, MN, USA). TRIzol reagent (Invitrogen, Carlsbad, CA, USA) was used for RNA isolation and molecular analyses. Primary antibodies against p-STAT3, STAT3, and β-actin were obtained from Cell Signaling Technology (Danvers, MA, USA), whereas enhanced chemiluminescence (ECL) reagents for protein detection were purchased from Bio-Rad Laboratories (Hercules, CA, USA). The purified peptide, WPN-15 (NGAIADQQPQRPNIV; molecular weight, 1620.77 Da), was chemically synthesized at a purity exceeding 95% by A&Pep Co., Ltd. (Cheongju, Republic of Korea) and subsequently used for all in vitro and in vivo experiments.

### 2.2. Preparation of Enzymatic Hydrolysates from Walleye Pollock Tail By-Products

**Figure 1:**
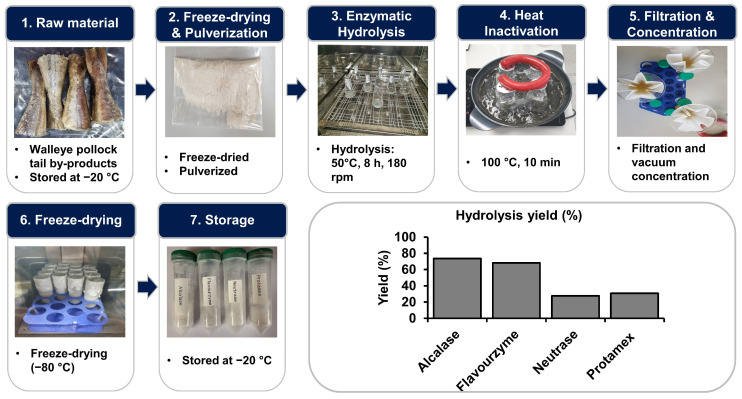
Workflow for the preparation of enzymatic hydrolysates from Walleye pollock tail by-products and comparison of hydrolysis yields.

Fresh walleye pollock tail by-products were thoroughly rinsed with distilled water, lyophilized, and ground into a fine powder before enzymatic hydrolysis. The powdered material was dispersed in 0.1 M phosphate buffer (pH 7.0) at a substrate-to-buffer ratio of 1:50 (*w*/*v*) and equilibrated at 50 °C for 30 min under constant agitation (150 rpm). Hydrolysis was subsequently carried out using four commercial food-grade proteases (Alcalase, Flavourzyme, Neutrase, and Protamex), each added at an enzyme-to-substrate ratio of 1:50 (*w*/*w*). The reaction mixtures were incubated at 50 °C for 8 h with continuous shaking at 180 rpm. Enzyme activity was terminated by heating the mixtures at 100 °C for 10 min. The resulting hydrolysates were clarified by filtration through Whatman No. 1 filter paper (Cytiva, Marlborough, MA, USA) to remove insoluble materials, concentrated under reduced pressure, and lyophilized to obtain peptide-rich hydrolysate powders. All hydrolysate samples were stored at −20 °C until subsequent analyses. A schematic overview of the hydrolysis procedure is shown in Figure 1. Hydrolysis yield was determined as the percentage of dried hydrolysate recovered relative to the initial dry weight of the substrate. Among the four enzymes evaluated, Alcalase and Protamex produced markedly higher hydrolysis yields (73.71% and 68.25%, respectively) than Flavourzyme (27.68%) and Neutrase (30.75%) (Figure 1). The anti-inflammatory potential of each hydrolysate was subsequently assessed by measuring nitric oxide (NO) production and cell viability in LPS-stimulated RAW 264.7 macrophages.

### 2.3. Cell Viability

RAW 264.7 murine macrophages were maintained in DMEM supplemented with 10% FBS and 1% penicillin-streptomycin under standard culture conditions (37 °C, 5% CO_2_). Cell viability was evaluated using the MTT colorimetric assay as previously described [26]. For the assay, cells were plated in 96-well plates at a density of 1 × 10^5^ cells per well and allowed to stabilize for 24 h before treatment. The cultures were subsequently exposed to enzymatic hydrolysates, purified fractions, or WPN-15 at the indicated concentrations for an additional 24 h. Following treatment, MTT solution (5 mg/mL) was added to each well, and the plates were incubated for 4 h to allow the formation of formazan crystals. The resulting crystals were dissolved in DMSO, after which absorbance was recorded at 540 nm using a microplate reader (xMARK, Bio-Rad, Hercules, CA, USA). Cell viability was calculated relative to untreated control cells and expressed as a percentage.

### 2.4. Nitric Oxide (NO) Assays

NO production was evaluated by determining nitrite concentrations in the culture supernatant using the Griess colorimetric assay, as previously reported by Green et al. [27]. RAW 264.7 macrophages were plated in 24-well culture plates (2 × 10^5^ cells/well) and cultured overnight before treatment. The cells were then exposed to enzymatic hydrolysates, purified fractions, or WPN-15 for 1 h prior to stimulation with LPS (100 ng/mL), followed by incubation for an additional 24 h. After incubation, 100 μL of culture supernatant was combined with an equal volume of Griess reagent and allowed to react at room temperature for 10 min. Absorbance was subsequently recorded at 540 nm using a microplate reader (xMARK, Bio-Rad, Hercules, CA, USA), and nitrite concentrations were quantified from a sodium nitrite calibration curve.

### 2.5. Activity-Guided Purification of Bioactive Peptides

Based on its superior anti-inflammatory activity, the Neutrase hydrolysate was selected for subsequent peptide purification. The hydrolysate was first separated using a 3500 Da molecular weight cut-off (MWCO) dialysis membrane (Spectrum Laboratories, Rancho Dominguez, CA, USA) for 24 h, after which the fractions retained inside and outside the membrane were collected individually, lyophilized, and screened for NO inhibitory activity. The most active fraction was dissolved in distilled water (5 mg/mL) and further purified by fast protein liquid chromatography-gel permeation chromatography (FPLC-GPC) using a Superdex^TM^ Peptide 10/300 GL column (GE Healthcare, Uppsala, Sweden). Distilled water was used as the mobile phase at a flow rate of 0.5 mL/min, and peptide elution was continuously monitored by measuring absorbance at 280 nm. Fractions exhibiting NO inhibitory activity were freeze-dried and subjected to reverse-phase high-performance liquid chromatography (RP-HPLC) using a ZORBAX SB-C18 column (4.6 × 250 mm, 5 μm; Agilent Technologies, Santa Clara, CA, USA). Peptide separation was achieved with a linear acetonitrile gradient containing 0.1% trifluoroacetic acid (TFA) at a flow rate of 1.0 mL/min while monitoring absorbance at 280 nm. The collected subfractions were subsequently lyophilized and used for peptide identification.

### 2.6. Identification of WPN-15

The purified peptide was structurally characterized by chromatographic and mass spectrometric analyses. Peptide purity was confirmed by analytical RP-HPLC using a ZORBAX SB-C18 column (4.6 × 250 mm, 5 μm; Agilent Technologies). Chromatographic separation was carried out with a gradient elution system consisting of water and acetonitrile containing 0.1% TFA, and peptide elution was monitored at 216 nm. The molecular weight of the purified peptide was determined by electrospray ionization mass spectrometry (ESI-MS) using an Agilent 6125B LC/MS system (Agilent Technologies, Santa Clara, CA, USA) operated in positive ion mode. The amino acid sequence was determined by protein sequencing analysis using a PPSQ-51A protein sequencer (Shimadzu, Kyoto, Japan) at the Korea Advanced Institute of Science and Technology (KAIST, Daejeon, Republic of Korea). For subsequent biological experiments, WPN-15 was chemically synthesized at a purity greater than 95%—by A&Pep Co., Ltd. (Cheongju, Republic of Korea).

### 2.7. DNCB-Induced AD Mouse Model

**Figure 2:**
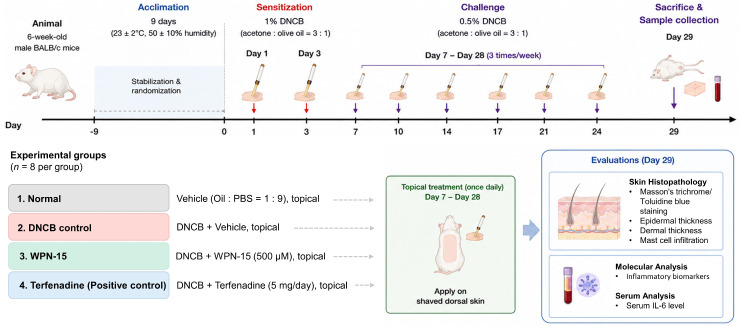
Schematic overview of the experimental design for the DNCB-induced atopic dermatitis mouse model and topical administration of WPN-15. Red arrows indicate sensitization with 1% DNCB (Days 1 and 3), and purple arrows indicate challenge with 0.5% DNCB (three times per week, Days 7–24). WPN-15 or terfenadine was topically administered once daily from Day 7 to Day 28. Animals were euthanized on Day 29, and skin tissues and blood samples were collected for histological, molecular, and serum analyses.

All animal procedures were performed in accordance with the guidelines approved by the Institutional Animal Care and Use Committee (IACUC) of Dankook University (Approval No. DKU-25-053 on 14 August 2025). Six-week-old male BALB/c mice were purchased from DBL Co., Ltd. (Eumseong, Republic of Korea) and maintained under controlled environmental conditions (23 ± 2 °C, 50 ± 10% relative humidity, and a 12 h light/dark cycle) with free access to standard chow and water. After a 9-day acclimation period, the animals were randomly allocated to four groups (*n* = 8 per group): Normal, DNCB control, WPN-15, and Terfenadine [25,28]. The experimental design is illustrated in Figure 2. AD was induced using DNCB dissolved in an acetone:olive oil mixture (3:1, *v*/*v*). The dorsal skin of the mice was shaved prior to sensitization. On Days 1 and 3, 1% DNCB was topically applied to the dorsal skin for sensitization. From Day 7 to Day 28, the mice were repeatedly challenged with 0.5% DNCB three times per week to maintain AD-like skin lesions. The Normal group received vehicle alone (oil:PBS = 1:9) instead of DNCB [26,27,28]. WPN-15 (500 μM) and terfenadine (5 mg/day) were topically applied once daily to the shaved dorsal skin from Day 7 to Day 28. The concentration of WPN-15 (500 μM) was selected based on our previous published study employing the same experimental model [25]. Terfenadine was used as a positive pharmacological control in this experimental model rather than as a standard therapy for clinical AD. The DNCB control group received vehicle alone. All treatment solutions were freshly prepared before application. On Day 29, the animals were euthanized, and skin tissues and blood samples were collected for subsequent analyses, as illustrated in Figure 2. Skin samples were processed for histopathological evaluation, including hematoxylin and eosin (H&E) staining and toluidine blue staining. Epidermal thickness, dermal thickness, and mast cell infiltration were evaluated. Serum samples were collected for the measurement of IL-6 levels. In addition, skin tissues were used for molecular analyses, including reverse transcription-polymerase chain reaction (RT-PCR) and Western blotting.

### 2.8. Evaluation of Dermatitis Severity Score

The severity of AD-like skin lesions was determined according to a modified clinical scoring system described previously [29]. The dorsal skin of each mouse was carefully inspected, and four representative clinical manifestations: erythema/hemorrhage, edema, excoriation/erosion, and dryness/scaling, were individually evaluated. Each parameter was assigned a score ranging from 0 to 3 (0 = none, 1 = mild, 2 = moderate, and 3 = severe). The total dermatitis severity score was obtained by summing the scores for all four parameters, yielding a maximum possible score of 12. Clinical evaluations were conducted under blinded conditions by two independent investigators to minimize observer bias.

### 2.9. Histopathological Analysis (H&E Staining)

To evaluate histopathological changes in the skin, dorsal skin tissues were collected on Day 29 and fixed in 10% neutral-buffered formalin at RT for 24 h. The tissues were processed through graded ethanol solutions for dehydration, cleared with xylene, and embedded in paraffin. Paraffin-embedded tissues were sectioned into 4 μm-thick slices using a microtome and mounted on glass slides. The sections were deparaffinized, rehydrated, and stained with H&E following standard histological protocols [30]. Histological images were captured using a light microscope (Olympus BX53, Olympus Corporation, Tokyo, Japan). Epidermal and dermal thicknesses were measured using ImageJ software 1.54 (National Institutes of Health, Bethesda, MD, USA). For each animal, measurements were obtained from at least five randomly selected microscopic fields, and the mean value was used for statistical analysis. Histopathological evaluation was conducted under blinded conditions.

### 2.10. Toluidine Blue Staining and Mast Cell Counting

To evaluate mast cell infiltration in AD skin lesions, paraffin-embedded dorsal skin sections were stained with toluidine blue following a previously reported method [31]. The tissue sections were first deparaffinized, rehydrated, and stained with 0.1% (*w*/*v*) toluidine blue solution for 2–3 min at RT. Following rinsing with distilled water, the sections were dehydrated, mounted, and examined under a light microscope.

Toluidine blue-positive mast cells, identified by their characteristic metachromatic dark blue–purple granules, were counted in five randomly chosen microscopic fields per section at a magnification of 200×. The number of mast cells are expressed as the average number of cells per microscopic field for each animal. All analyses were performed under blinded conditions to minimize observer bias.

### 2.11. Measurement of Serum IL-6 Levels

Serum IL-6 levels were quantified using a commercially available mouse IL-6 ELISA kit (R&D Systems) in accordance with the manufacturer’s instructions. Serum samples and IL-6 standards were sequentially dispensed into antibody-precoated microplate wells and incubated under the recommended conditions. After the washing steps, an enzyme-conjugated detection antibody was applied, followed by the addition of the substrate solution. The enzymatic reaction was terminated with stop solution, and optical density was recorded at 450 nm using a microplate reader. IL-6 concentrations were determined using the standard curve and expressed as pg/mL.

### 2.12. RT-PCR Analysis

Total RNA was extracted from dorsal skin tissues using TRIzol reagent (Invitrogen) following the manufacturer’s instructions. RNA concentration and purity were assessed spectrophotometrically, and 1 μg of total RNA was used for complementary DNA (cDNA) synthesis using a cDNA synthesis kit (Bioneer, Daejeon, Republic of Korea). The resulting cDNA served as the template for PCR amplification to determine the mRNA expression of *IL-1β* and *IL-6*. *Glyceraldehyde-3-phosphate dehydrogenase* (*GAPDH*) was used as an internal control. The primer sequences used were as follows: *IL-1β* forward, 5′-CATTGTGGCTGTGGAGAAG-3′ and reverse, 5′-ATCATCCCACGAGTCACAGA-3′; *IL-6* forward, 5′-AGTTGCCTTCTTGGGACTGA-3′ and reverse, 5′-TCCACGATTTCCCAGAGAAC-3′; and *GAPDH* forward, 5′-AGGTCGGTGTGAACGGATTTG-3′ and reverse, 5′-TGTAGACCATGTAGTTGAGGTCA-3′. PCR amplification was performed under the following thermal cycling conditionss: initial denaturation at 94 °C for 5 min, followed by 40 cycles of denaturation at 94 °C for 30 s, annealing at 56 °C for 60 s, and extension at 72 °C for 60 s. The amplified products were resolved on 2% agarose gels and subsequently visualized using a gel documentation system.

### 2.13. Western Blot Analysis

To examine the regulatory effect of WPN-15 on inflammatory signaling, dorsal skin tissues were homogenized in radioimmunoprecipitation assay (RIPA) buffer containing protease and phosphatase inhibitors. The lysates were centrifuged at 12,000× *g* for 15 min at 4 °C, and the supernatants were used for subsequent protein extraction and analysis. Protein concentrations were quantified using a bicinchoninic acid (BCA) protein assay kit (Thermo Fisher Scientific, Waltham, MA, USA). Equal amounts of protein (30 μg) were separated by sodium dodecyl sulfate-polyacrylamide gel electrophoresis (SDS-PAGE) and transferred onto polyvinylidene fluoride (PVDF) membranes. The membranes were blocked with 5% skim milk in Tris-buffered saline containing 0.1% Tween-20 (TBST) and incubated overnight at 4 °C with primary antibodies against p-STAT3, STAT3, and β-actin (Cell Signaling Technology). Following TBST washes, the membranes were incubated with horseradish peroxidase (HRP)-conjugated secondary antibodies for 1 h at RT. Protein bands were visualized using an ECL detection kit (Bio-Rad Laboratories) and quantified using ImageJ software. β-Actin was used as the internal loading control.

### 2.14. Statistical Analysis

All data are presented as the mean ± standard deviation (SD). Statistical analyses were conducted using GraphPad Prism software (version 10.0; GraphPad Software, San Diego, CA, USA). Differences among groups were evaluated using one-way analysis of variance (ANOVA) followed by Tukey’s post hoc multiple comparison test. A *p*-value < 0.05 was considered to indicate statistical significance. Groups labeled with different letters were considered significantly different (*p* < 0.05).

## 3. Results

### 3.1. Activity-Guided Purification of Anti-Inflammatory Peptides from Walleye Pollock Tail Hydrolysates

**Figure 3:**
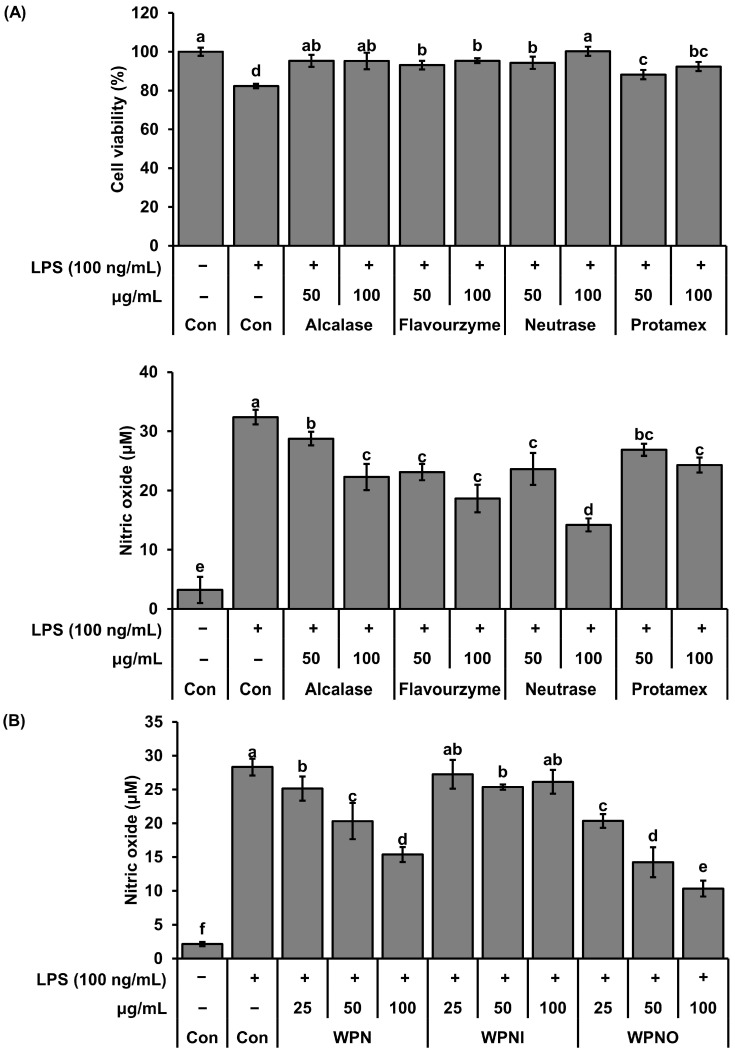

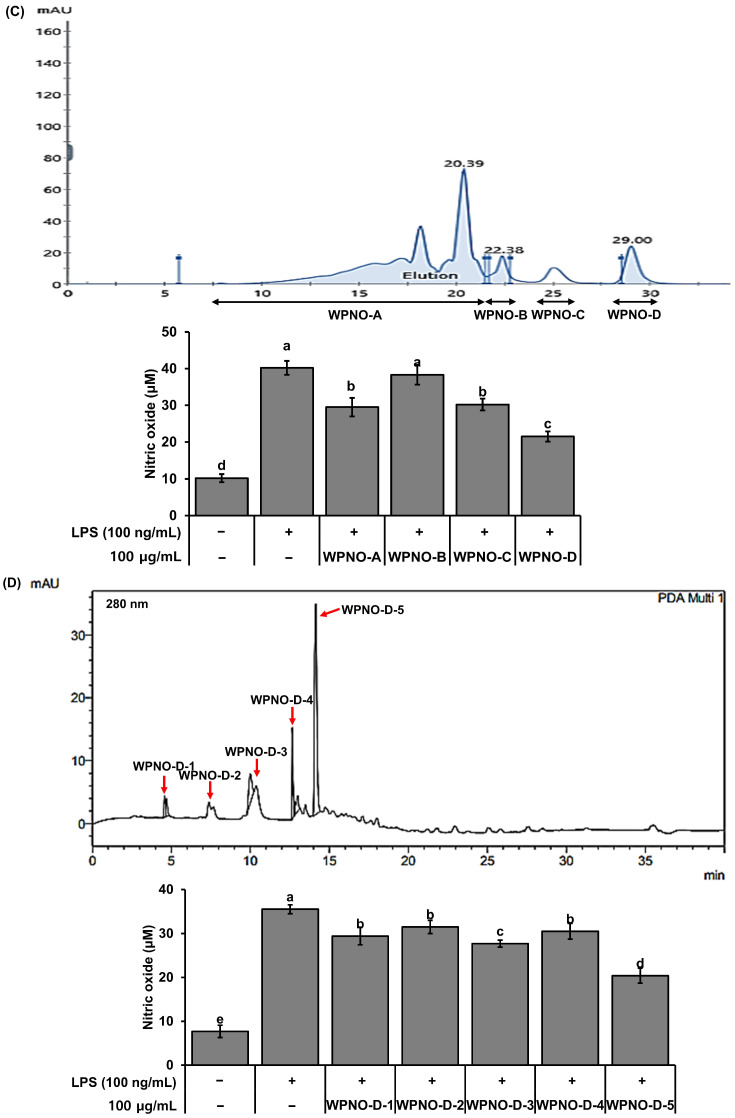
Sequential activity-guided isolation of the anti-inflammatory peptide WPN-15 from walleye pollock tail hydrolysates. (**A**) Effects of enzymatic hydrolysates generated with Alcalase, Flavourzyme, Neutrase, and Protamex on cell viability and NO production in LPS-stimulated RAW 264.7 macrophages. (**B**) NO inhibitory activity of fractions separated using a 3500 Da dialysis membrane. The low-molecular-weight fraction (WPNO, <3500 Da) showed the greatest inhibitory activity. (**C**) FPLC-GPC chromatogram of the WPNO fraction together with the NO inhibitory effects of the collected fractions (WPNO-A to WPNO-D). WPNO-D displayed the strongest activity. (**D**) RP-HPLC fractionation of WPNO-D and NO inhibitory activities of the resulting subfractions (WPNO-D-1 to WPNO-D-5). WPNO-D-5 produced the greatest reduction in NO production and was therefore chosen for structural identification. Data are presented as mean ± SD (*n* = 3). Groups denoted by different letters are significantly different (*p* < 0.05).

To identify anti-inflammatory peptides from walleye pollock tail by-products, hydrolysates prepared using four commercial proteases were initially evaluated for their effects on cell viability and NO production in LPS-stimulated RAW 264.7 macrophages (Figure 3A). Treatment with all hydrolysates maintained cell viability above 90% at both 50 and 100 μg/mL, indicating that none of the samples exhibited appreciable cytotoxicity under the experimental conditions. Among the tested hydrolysates, the Neutrase-derived hydrolysate produced the greatest reduction in NO production, decreasing nitrite levels from 32.5 μM in the LPS-treated control group to 14.2 μM at a concentration of 100 μg/mL. Based on this superior anti-inflammatory activity, the Neutrase hydrolysate was selected for subsequent purification procedures. The Neutrase hydrolysate was subsequently fractionated using a 3500 Da molecular weight cut-off dialysis membrane (Figure 3B). Following dialysis, the low-molecular-weight fraction collected outside the membrane (WPNO, <3500 Da) exhibited substantially stronger inhibition of NO production than both the unfractionated hydrolysate (WPN) and the retained fraction (WPNI). At 100 μg/mL, WPNO reduced NO production to approximately 10.1 μM, indicating that peptides with molecular weights below 3500 Da were primarily responsible for the observed anti-inflammatory activity. To further purify the active components, the WPNO fraction was subjected to fast protein liquid chromatography-gel permeation chromatography (FPLC-GPC) (Figure 3C), resulting in four major fractions designated as WPNO-A, WPNO-B, WPNO-C, and WPNO-D. Among these fractions, WPNO-D displayed the strongest suppression of LPS-induced NO production and was therefore selected for further purification. Subsequent separation of the WPNO-D fraction by RP-HPLC on a C18 column generated five distinct subfractions (WPNO-D-1 to WPNO-D-5) (Figure 3D). Comparative evaluation of their anti-inflammatory activities demonstrated that WPNO-D-5 exhibited the greatest inhibitory effect on NO production. Accordingly, WPNO-D-5 was selected for structural identification and subsequent biological characterization.

### 3.2. Identification and Characterization of WPN-15

**Figure 4:**
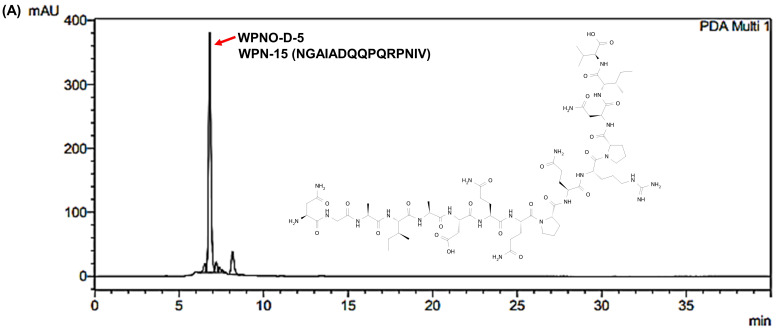

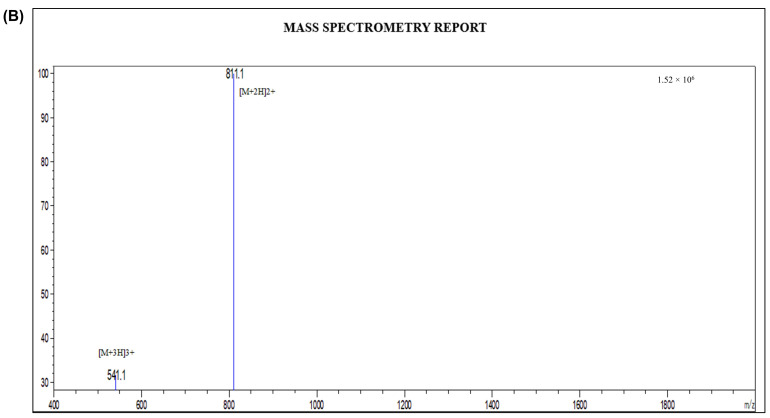
Identification and characterization of the purified peptide WPN-15. (**A**) Analytical RP-HPLC chromatogram of the purified fraction WPNO-D-5. A single dominant peak was observed, indicating the high purity of the isolated peptide. The amino acid sequence of the purified peptide was identified as NGAIADQQPQRPNIV (WPN-15), and its chemical structure is shown. (**B**) ESI-MS spectrum of WPN-15. The major ion peaks were detected at *m*/*z* 811.1 ([M + 2H]^2+^) and *m*/*z* 541.1 ([M + 3H]^3+^), corresponding to a calculated molecular weight of 1620.77 Da.

The most active fraction obtained through activity-guided purification, designated as WPNO-D-5, was subjected to further structural characterization. As shown in Figure 4A, analytical RP-HPLC analysis revealed a single dominant peak, indicating that the purified fraction possessed a high degree of purity. Amino acid sequence analysis identified the purified peptide as NGAIADQQPQRPNIV, which was designated as WPN-15. The molecular weight of WPN-15 was subsequently determined by electrospray ionization mass spectrometry (ESI-MS). As shown in Figure 4B, the mass spectrum exhibited major ion peaks at *m*/*z* 811.1 ([M + 2H]^2+)^ and *m*/*z* 541.1 ([M + 3H]^3+^). These ion signals corresponded to a calculated molecular weight of 1620.77 Da, confirming the identity of the purified peptide. Collectively, the chromatographic and mass spectrometric analyses verified that WPN-15 is a highly purified peptide consisting of 15 amino acid residues with a molecular weight of 1620.77 Da. Based on these results, WPN-15 was selected for subsequent in vivo evaluation in the DNCB-induced atopic dermatitis mouse model.

### 3.3. Effects of WPN-15 on Clinical Symptoms and Serum IL-6 Levels in DNCB-Induced AD Mice

**Figure 5:**
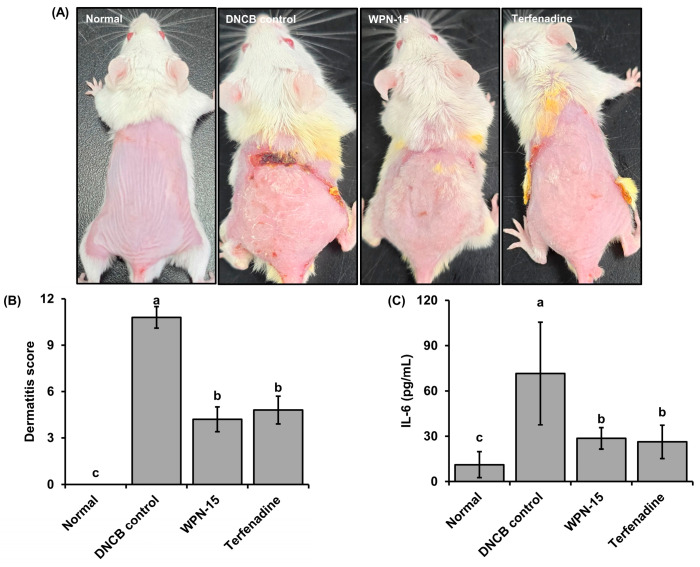
Effects of WPN-15 on the clinical manifestation sand inflammatory responses in DNCB-induced AD mice. (**A**) Representative images showing dorsal skin lesions in the Normal, DNCB control, WPN-15, and terfenadine-treated groups. (**B**) Dermatitis severity scores. (**C**) Serum IL-6 levels. Data are presented as mean ± SD (*n* = 8). Groups labeled with different letters differ significantly (*p* < 0.05).

The therapeutic effects of WPN-15 on DNCB-induced AD were evaluated by assessing clinical skin symptoms, dermatitis severity scores, and serum IL-6 levels. As illustrated in Figure 5A, repeated topical exposure to DNCB produced typical AD-like skin lesions, including erythema, edema, excoriation, scaling, and crust formation. In contrast, mice treated with WPN-15 exhibited marked improvement in these pathological skin manifestations, resulting in a visibly healthier skin appearance than that observed in the DNCB control group. The degree of improvement achieved with WPN-15 was comparable to that of the terfenadine-treated group. The macroscopic findings were further supported by quantitative analysis of dermatitis severity scores (Figure 5B). DNCB challenge significantly increased the dermatitis score relative to the Normal group, whereas topical application of WPN-15 significantly attenuated disease severity compared with the DNCB control group (*p* < 0.05). No statistically significant difference was detected between the WPN-15 and terfenadine groups, although both treatments produced significantly lower scores than the DNCB control group. A similar trend was observed for serum IL-6 concentrations (Figure 5C). DNCB treatment markedly elevated circulating IL-6 levels compared with those in the Normal group. Administration of WPN-15 significantly reduced this DNCB-induced increase in serum IL-6 (*p* < 0.05). Furthermore, serum IL-6 levels in the WPN-15-treated group were comparable to those in the terfenadine-treated group, and both treatment groups exhibited significantly lower IL-6 concentrations than the DNCB control group.

### 3.4. Histopathological Improvements Induced by WPN-15 in DNCB-Induced AD Mice

**Figure 6:**
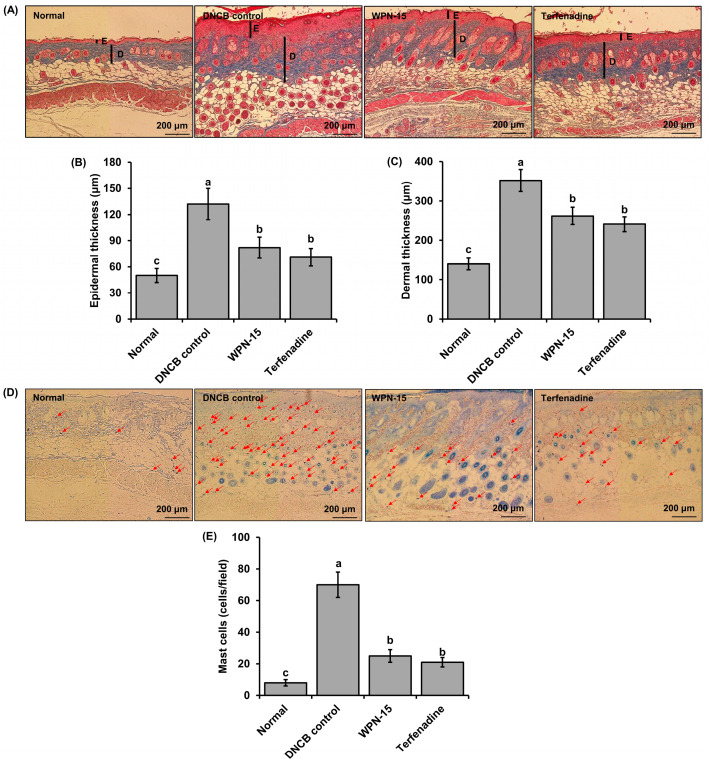
Histopathological effects of WPN-15 in DNCB-induced atopic dermatitis mice. (**A**) Representative H&E-stained images of dorsal skin tissues from the Normal, DNCB control, WPN-15, and terfenadine groups. E and D indicate the epidermis and dermis, respectively. (**B**) Measurement ofepidermal thickness. (**C**) Measurement ofdermal thickness. (**D**) Representative toluidine blue-stained dorsal skin sections illustrating mast cell infiltration (arrows). (**E**) Quantitative analysis of mast cell infiltration. Data are presented as mean ± SD (*n* = 8). Groups labeled with different letters differ significantly (*p* < 0.05). Scale bars = 200 μm.

To further investigate the protective effects of WPN-15 against DNCB-induced AD, histopathological changes in dorsal skin tissues were examined by H&E and toluidine blue staining. As presented in Figure 6A, DNCB treatment caused pronounced epidermal hyperplasia, dermal thickening, and inflammatory cell infiltration compared with the Normal group. These pathological alterations were markedly alleviated by topical application of WPN-15, resulting in skin morphology that closely resembled that of the terfenadine-treated mice. Morphometric analysis confirmed these histological observations (Figure 6B). Epidermal thickness was significantly greater in the DNCB control group than in the Normal group. Topical treatment with WPN-15 significantly decreased epidermal thickness compared with the DNCB control group (*p* < 0.05). No significant difference was detected between the WPN-15- and terfenadine-treated groups, whereas both treatments produced significantly lower epidermal thickness than the DNCB control group. A comparable pattern was observed for dermal thickness (Figure 6C). DNCB exposure markedly increased dermal thickness, whereas administration of WPN-15 significantly attenuated this pathological change (*p* < 0.05). The reduction achieved by WPN-15 was comparable to that observed following terfenadine treatment. Toluidine blue staining demonstrated extensive mast cell infiltration within the dermis of DNCB-treated mice (Figure 6D). Quantitative evaluation further revealed a significant increase in mast cell numbers in the DNCB control group compared with the Normal group (Figure 6E). Treatment with WPN-15 significantly decreased mast cell infiltration relative to the DNCB control group (*p* < 0.05). Similar to the findings for epidermal and dermal thickness, no significant difference was observed between the WPN-15 and terfenadine groups, while both groups exhibited significantly fewer mast cells than the DNCB control group. Overall, these results indicate that WPN-15 effectively mitigated DNCB-induced histopathological damage by suppressing epidermal hyperplasia, dermal thickening, and mast cell accumulation, thereby reducing the inflammatory changes associated with AD.

### 3.5. Effects of WPN-15 on Inflammatory Gene Expression and STAT3 Activation in DNCB-Induced AD Mice

**Figure 7:**
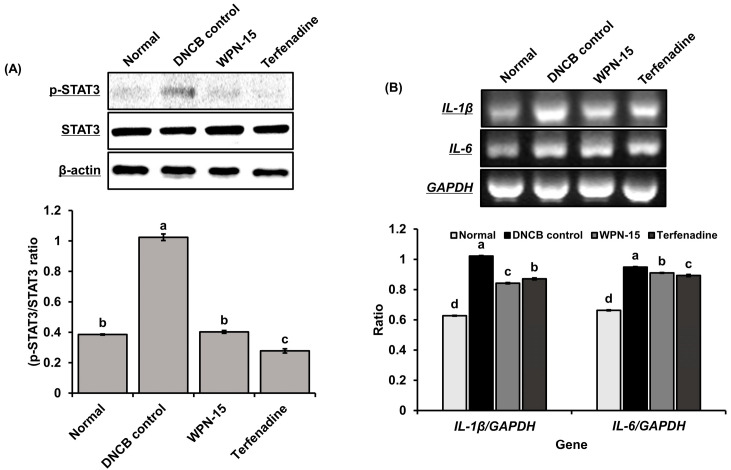
Effects of WPN-15 on inflammatory cytokine expression and STAT3 signaling in DNCB-induced atopic dermatitis mice. (**A**) Representative Western blot images showing p-STAT3, total STAT3, and β-actin protein expression, together with quantitative analysis of the p-STAT3/STAT3 ratio, in dorsal skin tissues. (**B**) Representative RT-PCR images and quantitative analysis of the relative mRNA expression of *IL-1β* and *IL-6*. Data are presented as mean ± SD (*n* = 3). Groups labeled with different letters differ significantly (*p* < 0.05).

To investigate the molecular mechanisms underlying the anti-inflammatory effects of WPN-15, the expression levels of inflammatory cytokines and STAT3 signaling-related proteins were analyzed in dorsal skin tissues obtained from DNCB-induced AD mice. As illustrated in Figure 7A, DNCB challenge markedly increased the phosphorylation of STAT3 compared with the Normal group, whereas total STAT3 protein expression showed no noticeable differences among the experimental groups. Densitometric analysis revealed a significant elevation of the p-STAT3/STAT3 ratio in the DNCB control group. Topical treatment with WPN-15 significantly suppressed STAT3 phosphorylation compared with the DNCB control (*p* < 0.05), bringing the p-STAT3/STAT3 ratio close to that observed in the Normal group. Terfenadine treatment resulted in the lowest p-STAT3/STAT3 ratio among all experimental groups. The transcriptional expression of the pro-inflammatory cytokines IL-1β and IL-6 was further evaluatedby RT-PCR (Figure 7B). DNCB exposure significantly upregulated the mRNA expression of both cytokines relative to the Normal group. Administration of WPN-15 significantly reduced the DNCB-induced elevation of IL-1β and IL-6. Statistical analysis demonstrated that IL-1β expression was highest in the DNCB control group, intermediate in the WPN-15-treated group, and significantly lower in the terfenadine-treated and Normal groups. A comparable trend was observed for IL-6 expression, with the highest transcript level detected in the DNCB control group, a significant reduction following WPN-15 treatment, and the lowest expression in the terfenadine-treated and Normal groups. Collectively, these results suggest that WPN-15 alleviates DNCB-induced skin inflammation by suppressing pro-inflammatory cytokine expression and inhibiting STAT3 activation.

## 4. Discussion

AD is a multifactorial inflammatory skin disease in which epidermal barrier impairment, immune imbalance, excessive inflammatory cytokine production, and infiltration of inflammatory cells collectively contribute to disease development and progression [32,33]. Although biologics and JAK inhibitors have considerably expanded therapeutic options for AD, the identification of safer, naturally derived therapeutic agents remains an important area of investigation [34,35,36,37,38]. In this study, we successfully identified a novel marine-derived peptide, WPN-15 (NGAIADQQPQRPNIV), from enzymatically hydrolyzed walleye pollock tail by-products through an activity-guided purification strategy (Figure 3 and Figure 4). Its therapeutic potential was subsequently validated in a DNCB-induced mouse model of AD.

Marine-derived peptides have attracted considerable interest as promising functional ingredients for pharmaceutical and nutraceutical applications because of their broad spectrum of biological activities, including antioxidant, anti-inflammatory, antimicrobial, and immunomodulatory activities [39]. In parallel, protein-rich by-products generated during fish processing are increasingly being recognized as sustainable raw materials for the development of bioactive peptides [40]. In the present study, WPN-15 was identified from Neutrase hydrolysates of walleye pollock tail by-products using an activity-guided purification approach (Figure 3). The fraction containing low-molecular-weight peptides (<3500 Da) exhibited the strongest inhibition of NO production, suggesting that peptides within this molecular weight range were primarily responsible for the observed anti-inflammatory activity [41]. These findings are consistent with previous studies reporting that low-molecular-weight peptides often exhibit superior biological activity because of their enhanced bioavailability and improved tissue penetration [42]. The slightly stronger activity observed in the unfractionated low-molecular-weight peptide mixture than in the purified peptide may reflect synergistic interactions among multiple bioactive peptide components that were partially lost during purification. RAW264.7 macrophages were used as an initial screening platform to identify active peptide fractions before in vivo validation. Although keratinocyte-based models are widely used in AD research, future studies employing HaCaT or primary keratinocytes will be valuable for clarifying the direct effects of WPN-15 on epidermal cells.

The DNCB-induced mouse model closely reproduces many pathological characteristics of human AD, including erythema, edema, epidermal hyperplasia, inflammatory cell infiltration, and excessive cytokine production [43]. Consistent with these pathological features, repeated DNCB application produced severe AD-like skin lesions, whereas topical administration of WPN-15 markedly improved the gross appearance of the affected skin (Figure 5A) [44]. These macroscopic observations were supported by quantitative analysis showing a significant reduction in dermatitis severity scores following WPN-15 treatment (Figure 5B) [45]. Collectively, these findings indicate that WPN-15 effectively ameliorated both the clinical manifestations and inflammatory responses associated with experimental AD.

Among the numerous inflammatory mediators involved in AD, IL-6 is recognized as a central cytokine that promotes immune cell recruitment, keratinocyte activation, and chronic inflammatory responses through activation of downstream signaling pathways such as JAK/STAT [8,46,47]. Consistent with this mechanism, serum IL-6 concentrations were markedly elevated following DNCB exposure but were significantly reduced after topical administration of WPN-15 (Figure 5C). The observed decrease in circulating IL-6 suggests that WPN-15 may suppress not only local cutaneous inflammation but also systemic inflammatory responses associated with AD. Similar reductions in IL-6 have been reported for other anti-atopic agents in DNCB-induced experimental models [25]. Therefore, inhibition of IL-6-mediated inflammatory signaling is likely to represent one of the major mechanisms responsible for the therapeutic activity of WPN-15.

Histological evaluation provided additional evidence supporting the protective effects of WPN-15. DNCB challenge induced pronounced epidermal hyperplasia and dermal thickening, both of which are characteristic pathological features of chronic AD lesions (Figure 6A). Quantitative morphometric analysis confirmed significant increases in epidermal and dermal thickness in the DNCB control group (Figure 6B,C), whereas WPN-15 treatment significantly attenuated both parameters. These findings indicate that WPN-15 effectively suppresses chronic inflammatory remodeling within the skin. Previous investigations have similarly demonstrated that persistent immune activation disrupts epidermal homeostasis and promotes epidermal hyperplasia and inflammatory cell infiltration in AD [48]. Moreover, several marine-derived natural products have been reported to improve these histopathological abnormalities in experimental AD models [49,50]. Accordingly, restoration of normal epidermal and dermal architecture following WPN-15 treatment further supports its therapeutic potential for chronic inflammatory skin disorders.

Mast cells are recognized as major contributors to allergic skin inflammation by releasing histamine, cytokines, proteases, and other inflammatory mediators, which amplify pruritus and local inflammatory responses [51]. In agreement with this concept, extensive mast cell infiltration was observed in DNCB-treated skin tissues, whereas WPN-15 markedly reduced mast cell accumulation (Figure 6D,E). Reduced mast cell infiltration is likely to have contributed to the improvement in both histopathological alterations and clinical symptoms observed following WPN-15 treatment. Similar observations have been reported for other therapeutic agents that alleviate experimental AD by limiting mast cell recruitment and activation [52].

Another important finding of this study is that WPN-15 effectively inhibited activation of the STAT3 signaling pathway. Recent studies have demonstrated that the IL-6/JAK/STAT3 signaling axis serves as a critical regulator of AD progression by enhancing inflammatory cytokine production, keratinocyte activation, epidermal hyperplasia, and immune dysregulation [46,47,53]. Consistent with this mechanism, DNCB markedly increased STAT3 phosphorylation, whereas WPN-15 significantly reduced the p-STAT3/STAT3 ratio (Figure 7A). In parallel, RT-PCR analysis demonstrated significant downregulation of IL-1β and IL-6 expression following WPN-15 treatment (Figure 7B). Because IL-6 functions as a major upstream activator of STAT3, the concurrent reduction in IL-6 expression and STAT3 phosphorylation supports the possibility that modulation of the IL-6/JAK/STAT3 signaling cascade contributes to the anti-inflammatory effects of WPN-15. However, the present findings demonstrate an association rather than direct pathway inhibition, and additional mechanistic studies are required to identify the primary molecular target of WPN-15.

Compared with our previous study, which isolated a peptide through antioxidant activity-guided purification [25], the present investigation employed an anti-inflammatory activity-guided strategy and identified a structurally distinct peptide with significant anti-atopic efficacy in vivo. Nevertheless, several limitations should be considered. Although WPN-15 reduced IL-6 expression and STAT3 phosphorylation, the direct molecular target responsible for these effects remains unknown. Future studies employing pathway-specific inhibitors, gene knockdown, or gene-editing approaches will be necessary to establish a causal relationship between WPN-15 and IL-6/JAK/STAT3 signaling. Furthermore, additional AD-related biomarkers, including Th2- and Th17-associated cytokines, serum IgE, and chemokines, should be evaluated to provide a more comprehensive understanding of its immunomodulatory activity. The present study also did not directly assess epidermal barrier function. Therefore, future investigations should examine barrier-associated proteins such as filaggrin, involucrin, loricrin, and claudin-1 together with functional barrier recovery. Finally, pharmacokinetic characteristics, skin permeability, long-term safety, and efficacy in human skin models and clinical studies should be investigated to facilitate clinical translation. The present study should be considered a preliminary proof-of-concept study designed to evaluate the initial therapeutic efficacy of WPN-15 in a well-established DNCB-induced atopic dermatitis model. Therefore, sex was not included as an experimental variable, and the exclusive use of male mice may introduce sex bias. Future experiments should evaluate potential sex-dependent differences in therapeutic efficacy and underlying mechanisms by including both male and female animals, in accordance with current recommendations to consider sex as a biological variable in preclinical research. Collectively, these investigations will further clarify the molecular mechanisms of WPN-15 and support its development as a novel marine-derived peptide for the treatment of inflammatory skin diseases.

## 5. Conclusions

In conclusion, WPN-15 (NGAIADQQPQRPNIV), identified as a novel peptide from walleye pollock tail by-products using an activity-guided purification approach, exhibited significant protective effects against AD in a DNCB-induced mouse model. WPN-15 ameliorated clinical skin symptoms and reduced dermatitis severity scores, while attenuating epidermal hyperplasia, dermal thickening, and mast cell infiltration. Furthermore, WPN-15 downregulated the expression of IL-1β and IL-6 and inhibited STAT3 phosphorylation. These findings suggest that the anti-inflammatory activity of WPN-15 is mediated, at least in part, through regulation of the IL-6/STAT3 signaling pathway. Overall, our findings demonstrate that walleye pollock tail by-products are a sustainable and valuable source of bioactive peptides and support the potential application of WPN-15 as a marine-derived peptide candidate for the prevention and management of AD.

## Data Availability

All data generated or analyzed during this study were included in this article.

## Abbreviations

The following abbreviations are used in this manuscript:

AD	Atopic dermatitis
DNCB	2,4-Dinitrochlorobenzene
MWCO	Molecular weight cut-off
WPN	Walleye pollock Neutrase hydrolysate
WPNI	Fraction retained inside the dialysis membrane (>3500 Da)
WPNO	Fraction outside the dialysis membrane (<3500 Da)
WPN-15	Bioactive peptide (NGAIADQQPQRPNIV) isolated from walleye pollock tail by-products
